# Targeting protein tyrosine phosphatase σ after myocardial infarction restores cardiac sympathetic innervation and prevents arrhythmias

**DOI:** 10.1038/ncomms7235

**Published:** 2015-02-02

**Authors:** R. T. Gardner, L. Wang, B. T. Lang, J. M. Cregg, C. L. Dunbar, W. R. Woodward, J. Silver, C. M. Ripplinger, B. A. Habecker

**Affiliations:** 1 Department of Physiology and Pharmacology, Neuroscience Graduate Program, Oregon Health and Science University, Portland, Oregon 97239, USA; 2 Department of Neurology, Oregon Health and Science University, Portland, Oregon 97239, USA; 3 Department of Pharmacology, University of California, Davis, California 95616, USA; 4 Department of Neurosciences, Case Western Reserve University, Cleveland, Ohio 44106, USA

## Abstract

Millions of people suffer a myocardial infarction (MI) every year, and those who survive have increased risk of arrhythmias and sudden cardiac death. Recent clinical studies have identified sympathetic denervation as a predictor of increased arrhythmia susceptibility. Chondroitin sulfate proteoglycans present in the cardiac scar after MI prevent sympathetic reinnervation by binding the neuronal protein tyrosine phosphatase receptor σ (PTPσ). Here we show that the absence of PTPσ, or pharmacologic modulation of PTPσ by the novel intracellular sigma peptide (ISP) beginning 3 days after injury, restores sympathetic innervation to the scar and markedly reduces arrhythmia susceptibility. Using optical mapping we observe increased dispersion of action potential duration, supersensitivity to β-adrenergic receptor stimulation and Ca^2+^ mishandling following MI. Sympathetic reinnervation prevents these changes and renders hearts remarkably resistant to induced arrhythmias.

## Abstract (web summary)

Chondroitin sulfate proteoglycans (CSP) in the myocardial scar inhibit the tissue’s reinnervation, rendering it prone to arrhythmia. Here the authors show that blocking the activity of the CSP receptor, protein tyrosine phosphatase receptor σ, promotes scar reinnervation and prevents arrhythmia in mice.

## Graphical abstract

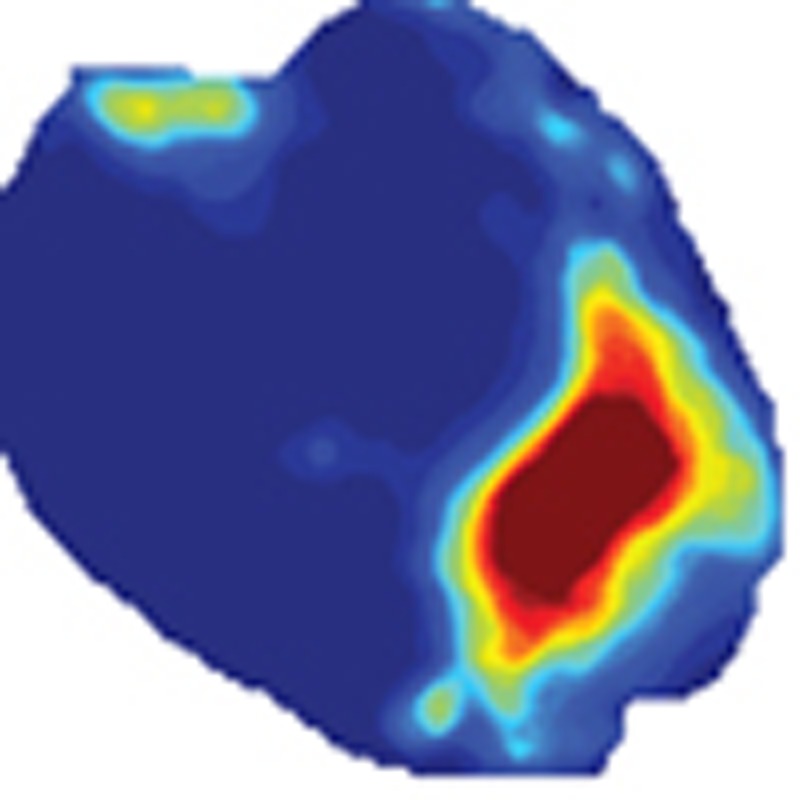

Survivors of myocardial infarction (MI) remain at high risk for cardiac arrhythmias and sudden cardiac death^1^. The infarct, or scar, generates an anatomical substrate that promotes re-entrant arrhythmias^2^, and numerous studies indicate that altered sympathetic neurotransmission in the heart also plays a key role in the onset of post-infarct cardiac arrhythmias^3^,^4^,^5^,^6^,^7^. Norepinephrine (NE) released from sympathetic nerves activates cardiac β-adrenergic receptors (β-AR) to modulate myocyte repolarization by altering transmembrane currents and Ca^2+^ homeostasis^8^,^9^,^10^, and simply disrupting the normal organization of sympathetic innervation in an otherwise healthy heart is arrhythmogenic^11^,^12^. Cardiac sympathetic function is altered in a region-specific manner following MI, and studies in animals and humans reveal denervation of the infarct and adjacent, viable (peri-infarct) myocardium^13^,^14^,^15^,^16^,^17^. Three recent studies in patients with implanted cardioverter defibrillators (ICDs) suggest that the amount of sympathetic denervation after MI predicts the probability of serious ventricular arrhythmias^18^,^19^,^20^. A detailed electrical mapping study in intact human hearts revealed that sympathetic denervation of the normal myocardium adjacent to the scar resulted in β-AR agonist supersensitivity and increased dispersion of repolarization that was arrhythmogenic^21^. These studies and others led to the model that inappropriate heterogeneity of sympathetic transmission across the left ventricle, and subsequent electrical remodelling of cardiac myocytes, is a major contributor to post-infarct arrhythmias in humans^22^.

The observation that the denervated myocardium adjacent to the infarct contributes to the generation of post-infarct arrhythmias^21^ was especially interesting to us because chondroitin sulfate proteoglycans (CSPGs) in the cardiac scar prevent reinnervation of the infarct and the adjacent myocardium by sympathetic axons^23^. Although axons sprout and regenerate towards the scar^24^, they are stopped near the outer edge of the infarct by CSPGs. In the absence of the CSPG receptor, protein tyrosine phosphatase receptor σ (PTPσ), sympathetic axons fully reinnervate undamaged peri-infarct tissue and hyperinnervate the infarct^23^. Given the clinical significance of sympathetic denervation after MI^18^,^19^,^20^,^21^, we were interested to determine whether restoring sympathetic innervation to the infarct and surrounding myocardium altered post-MI arrhythmia susceptibility.

We targeted PTPσ using both genetic and pharmacologic approaches in order to promote reinnervation of the infarct, and used electrocardiogram (ECG) telemetry to examine arrhythmia susceptibility. Transmembrane potential (*V*_m_) and intracellular Ca^2+^ dynamics were assessed using *ex vivo* optical mapping in order to investigate the mechanisms underlying changes in arrhythmia susceptibility. MI caused dispersion of action potential duration (APD), supersensitivity to β-AR stimulation and Ca^2+^ mishandling. Restoring sympathetic innervation to the infarct and the surrounding tissue decreased arrhythmia susceptibility and normalized cardiac electrophysiology and Ca^2+^ dynamics, despite the presence of a scar.

## Results

### Targeting PTPσ restores innervation after MI and prevents arrhythmias

**Figure 1:**
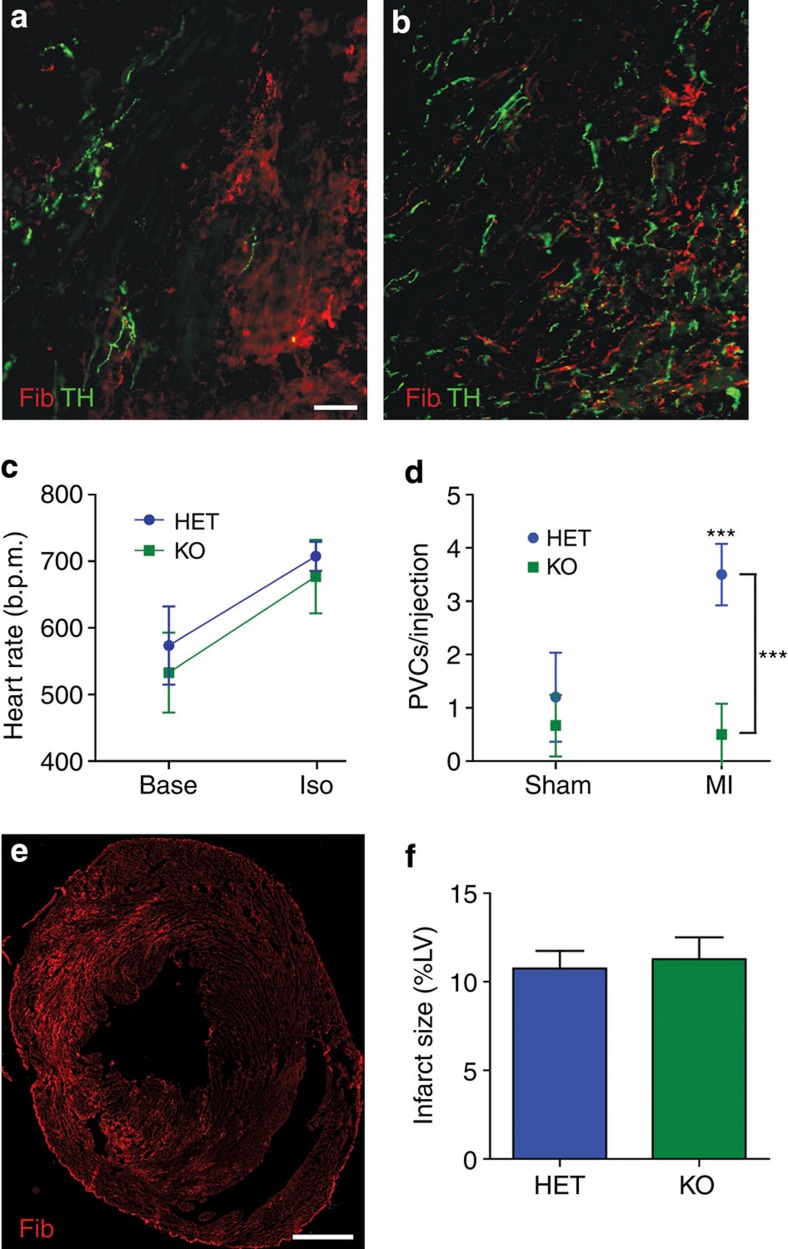
Absence of PTPσ restores innervation to the infarcted myocardium and prevents arrhythmias. Heart sections from HET (**a**) and KO (**b**) mice were stained for TH (green) to identify sympathetic nerve fibres and fibrinogen (red) to identify the infarct. Scale bar, 100 μm. (**c**) Heart rate was similar in both genotypes before (Base) and after (ISO) 10-μg ISO injection (mean±s.e.m., *n*=8 per genotype). (**d**) ISO induced comparable levels of PVCs in conscious Sham HET and KO mice; however, ISO induced significantly more PVCs in infarcted HET mice compared with KO mice and to Sham HET mice (mean±s.e.m., *n*=4 per group, ***P<0.001 versus HET sham and KO MI; two-way ANOVA with Bonferroni post test). (**e**) Fibrinogen-stained section after MI showing a transmural infarct after a 35-min occlusion. Scale bar, 500 μm. (**f**) Quantification of infarct size in HET and KO hearts following 35 min of occlusion (mean±s.e.m., *n*=4 per group; *t*-test, N.S. *P*>0.05).

We previously observed^23^ that CSPGs generated in the cardiac scar after ischaemia-reperfusion (I–R) prevented reinnervation of the infarct (Fig. 1a) despite high levels of nerve growth factor in the scar. The infarct becomes hyperinnervated in animals lacking the CSPG receptor PTPσ^23^ (Fig. 1b), confirming the crucial role for PTPσ in sympathetic denervation after MI. Since cardiac denervation is linked to risk for arrhythmia and cardiac arrest in human studies^18^,^19^,^20^,^21^, we asked whether restoring sympathetic innervation to the infarct and surrounding myocardium affected arrhythmia susceptibility. Control mice heterozygous for PTPσ (*ptprs*^+/−^; HET) and mice lacking PTPσ (*ptprs*^−/−^; KO) were implanted with ECG telemetry transmitters and then subjected to sham or MI surgery. Ten days after surgery, the mice were injected with 10 μg of the beta agonist isoproterenol (ISO), which has also been used to induce arrhythmias in the clinical setting^25^. ISO stimulated comparable increases in heart rate in all mice (Fig. 1c), but stimulated few premature ventricular complexes (PVCs) in sham mice of either genotype (Fig. 1d). In contrast, the arrhythmia response in post-MI animals differed on the basis of the innervation status of the infarct. ISO stimulated a significant number of PVCs in HET mice with denervated infarcts (Fig. 1d); however, KO mice with innervated infarcts were resistant to ISO-induced arrhythmias, having the same number of PVCs as sham animals (Fig. 1d). Infarcts were generally transmural (Fig. 1e), and infarct size was the same in both genotypes (Fig. 1f) indicating that the difference in arrhythmia susceptibility could not be explained by scar size.

**Figure 2:**
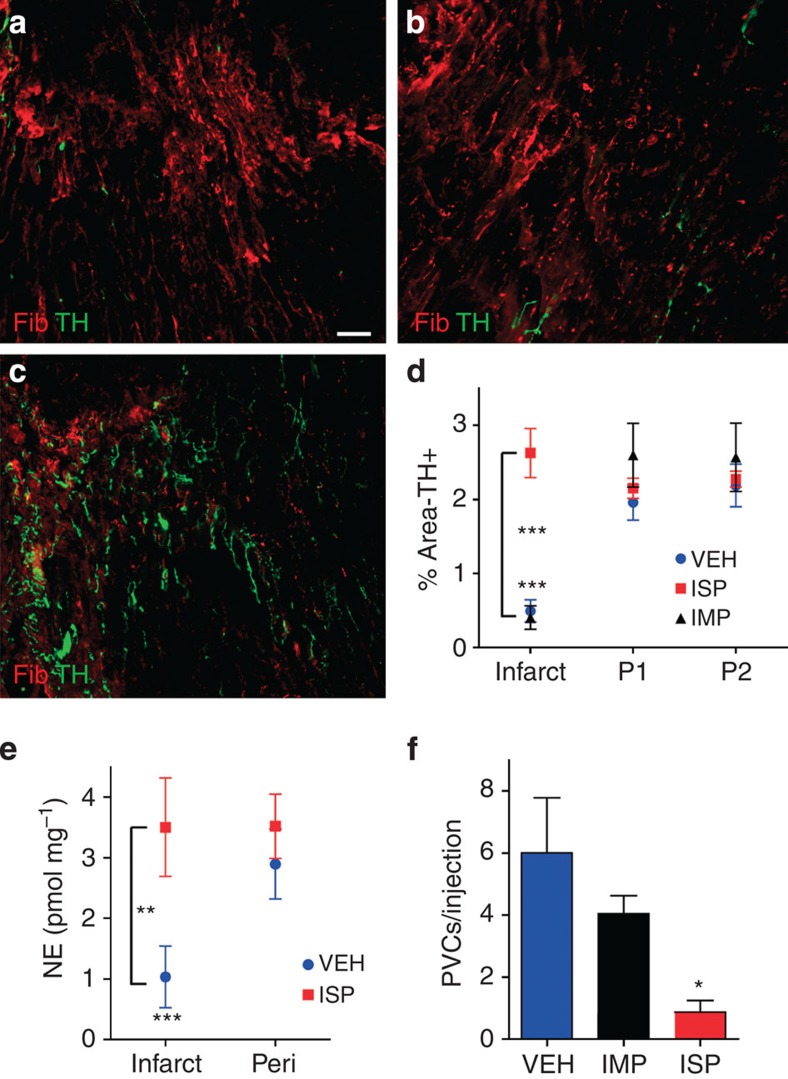
ISP promotes sympathetic reinnervation of the infarct and decreases arrhythmias. (**a**–**c**) Representative images of infarcted LV from mice treated with vehicle (**a**), IMP (**b**) or ISP (**c**) 14 days after MI. Sections were stained for TH (green) to identify sympathetic nerve fibres and fibrinogen (red) to identify the infarct. ISP treatment resulted in extensive sympathetic reinnervation of the infarct. Scale bar, 100 μm. (**d**) Quantification of TH+ fibre density within the infarct, the area immediately adjacent to the infarct (P1) and distal peri-infarct myocardium (P2; 540 μm from infarct) 14 day post-MI (mean±s.e.m., *n*=5 per group; ***P<0.001 by two-way ANOVA with Bonerroni post test). (**e**) NE content in the infarct and peri-infarct LV (P1 and P2 combined; mean±s.e.m.; *n*=5; **P<0.01, ***P<0.001 by two-way ANOVA with Bonerroni post test). (**f**) ISO induced PVCs in conscious mice 14 days after MI (mean±s.e.m., *n*=4–5; *P<0.05 versus vehicle and IMP by ANOVA with Tukey post test). Vehicle and IMP groups are not significantly different.

To confirm that reinnervation of the infarct and surrounding tissue decreased arrhythmia susceptibility, we sought to restore innervation in mice expressing normal levels of PTPσ. In order to promote regeneration of sympathetic axons through the CSPG-rich cardiac scar, we utilized ISP, developed by Lang and Silver^26^ to target the intracellular dimerization domain of PTPσ. ISP restored growth of the central nervous system axons through CSPGs *in vitro*, and systemic injections of ISP enhanced axon sprouting through CSPGs after spinal cord injury *in vivo*^26^. We asked whether targeting PTPσ with ISP could restore sympathetic axon regeneration into the cardiac scar. Wild-type (*ptprs^+/+^*) mice were implanted with ECG telemetry transmitters, and a week later subjected to I–R surgery. Beginning 3 days after MI, when the infarct was fully denervated^24^, mice were injected daily (intraperitoneal (IP)) with vehicle (5% dimethyl sulfoxide (DMSO)/Saline), ISP (10 μmol; 44 μg) or IMP (10 μmol; 42 μg) as a negative control. IMP targets the receptor PTPμ, which is not present in sympathetic neurons. Fourteen days after the MI surgery, the mice were treated with ISO to mimic circulating catecholamines and provoke arrhythmias, and hearts were collected for analysis of sympathetic innervation. Animals treated with either vehicle or IMP had denervated infarcts (Fig. 2a,b,d), whereas animals treated with ISP had normal levels of sympathetic innervation throughout the left ventricle, including the infarct (Fig. 2c,d). Thus, daily ISP injections beginning 3 days after the injury, when denervation was well established, allowed robust regeneration of sympathetic axons into the CSPG-laden cardiac scar.

Several studies indicate that newly regenerating sympathetic axons in the damaged heart have low levels of NE^27^,^28^, likely because of local depletion of Tyrosine Hydroxylase (TH) by inflammatory cytokines^29^. The cardiac scar in the mouse heart is mature by 10–12 days after reperfusion, and acute inflammation has resolved^30^. Therefore, we quantified NE levels in the infarct and peri-infarct myocardium 14 days after surgery to determine whether the axons that had reinnervated the infarct and surrounding myocardium had normal NE levels. NE content was low in the denervated infarct of vehicle-treated animals, but was normal in the innervated infarct of ISP-treated animals (Fig. 2e). NE content in the undamaged portion of the left ventricle was similar in both treatment groups (Fig. 2e), consistent with identical innervation densities (Fig. 2d). Likewise, NE content in the undamaged right ventricle was similar in both groups (Veh versus ISP; 5.50±0.70 versus 6.38 ±0.81 pmol mg^−1^; *P*=0.43). These data indicate that restoring innervation to the infarct with ISP normalizes NE levels across the left ventricle by 2 weeks after MI.

Since regional sympathetic denervation is thought to be an important source of post-MI arrhythmia susceptibility in humans, we quantified ISO-induced arrhythmias 14 days after MI in mice treated with vehicle, IMP or ISP. ISO triggered significantly fewer PVCs in ISP-treated mice, which had innervated infarcts and normal NE content, than in the vehicle or IMP-treated groups, which had denervated infarcts and low NE content (Fig. 2f). These data suggest that restoring sympathetic transmission throughout the infarcted left ventricle, even days after denervation is established, is sufficient to decrease arrhythmia susceptibility.

### Reinnervation limits dispersion of APD and β-AR supersensitivity

Activation of cardiac β receptors decreases myocyte APD by changing ion channel activity and Ca^2+^ handling to allow for adaptation to fast heart rates^8^,^9^,^10^. In order to assess directly the impact of infarct reinnervation on cardiac electrophysiology, we used optical mapping to simultaneously image intracellular Ca^2+^ and *V*_m_ in Langendorff-perfused isolated hearts. PTP KO mice were used to examine the effect of reinnervation on cardiac electrophysiology, with *ptprs*^+/−^ (HET) mice serving as denervated controls.

**Figure 3:**
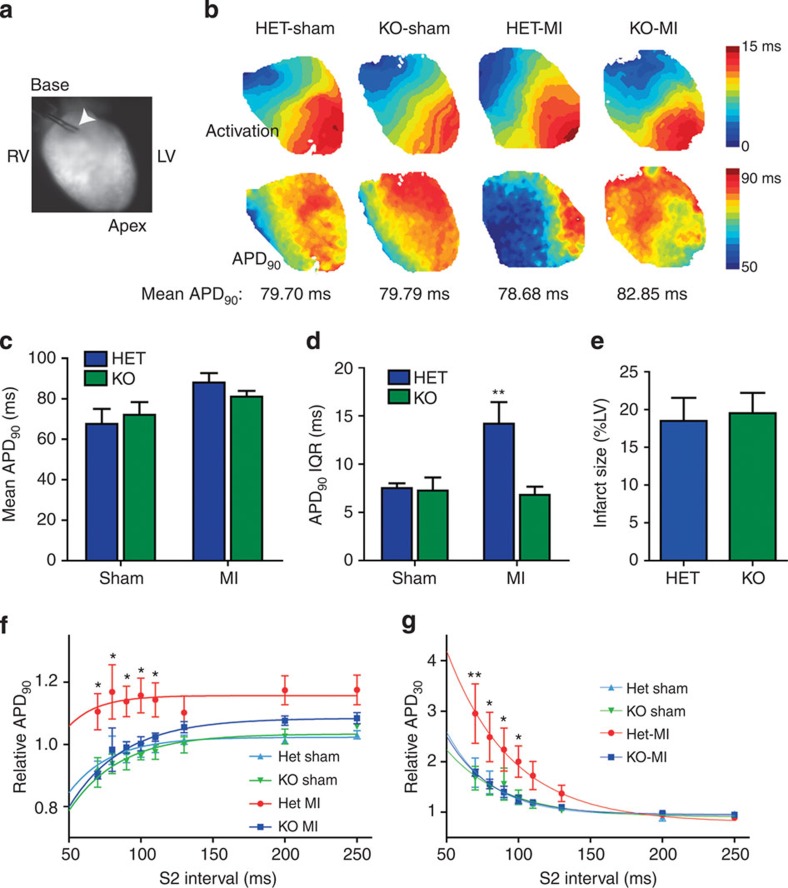
Sympathetic innervation of the infarct reduces dispersion of APD. (**a**) Image of a heart prepared for mapping. All maps are represented in this same orientation. White arrow head indicates stimulating electrodes; infarct region is in the apical LV. (**b**) Activation maps of transmembrane potential (*V*_m_) show propagation of a stimulus depolarization event spreading from the base to apex of the heart (top), and representative maps of APD_90_ (bottom). (**c**) The mean APD_90_ is similar in all groups (mean±s.e.m.; *n*=4–5 hearts per group). (**d**) IQR of APD_90_, a measure of APD dispersion (mean±s.e.m.; *n*=4–5, **P<0.01 by two-way ANOVA with Bonferroni post test). Hearts with denervated infarcts (HET MI) have increased APD dispersion compared with sham hearts, while hearts with innervated infarcts (KO MI) do not. (**e**) Infarct size in HET and KO hearts following 45 min of occlusion (mean±s.e.m., *n*=5 per group). Restitution curves of (**f**) late repolarization (APD_90_) and (**g**) early repolarization (APD_30_; mean±s.e.m., *n*=4–5; **P<0.01, *P<0.05 versus all other groups by two-way ANOVA with Bonferroni post test).

In order to compare baseline electrical properties, a pacing electrode was positioned on the base of the LV epicardium (Fig. 3a), and APD at 90% repolarization (APD_90_) was assessed at a pacing interval of 150 ms (400 b.p.m. heart rate). Figure 3b (top) shows a representative example of action potential propagation across a heart from each group: HET Sham, KO Sham, HET MI and KO MI. Figure 3b (bottom) shows a representative map of APD_90_ throughout each heart. Quantification of the mean APD_90_ revealed that it was similar across all four groups (Fig. 3c). However, it is clear that the denervated HET MI heart exhibits greater extremes of APD_90_ than the other hearts. The interquartile range (IQR) is a measure of APD_90_ variability, or dispersion, across an individual heart. IQR was significantly higher in denervated HET MI hearts compared with all other groups (Fig. 3d), consistent with increased dispersion of APD after MI in human studies^21^. Analysis of regional APD revealed a significant difference between the denervated infarct and normal myocardium (Supplementary Fig. 1). Surprisingly, sympathetic reinnervation of the infarct in KO hearts prevented an increase in APD dispersion following MI (Fig. 3d), even though the infarct size in the KO MI group was identical to that in the HET MI group (Fig. 3e).

Cycle length-dependent changes in APD (known as APD restitution) have also been implicated in arrhythmia generation^31^; therefore, we generated APD restitution curves using the pacing interval of 150 ms (S1) followed by a single extrastimulus (S2), with consecutively shorter S2 intervals. As expected, conduction velocity (Supplementary Fig. 2) and APD_90_ decreased with shorter S2 intervals in all hearts (Fig. 3f), while APD_30_ increased (Fig. 3g), consistent with previous studies of these properties in the mouse heart^32^. Interestingly, the HET MI hearts exhibited limited APD_90_ shortening compared with the other groups (Fig. 3f), and early repolarization (APD_30_) was blunted in those hearts at shorter S2 intervals (Fig. 3g). The increased APD_30_ observed in HET MI hearts is consistent with the reduction in transient outward K^+^ current (*I*_to_) seen after MI or sympathetic denervation^33^,^34^. However, KO MI hearts with sympathetic reinnervation exhibited APD restitution properties that were indistinguishable from sham hearts.

**Figure 4:**
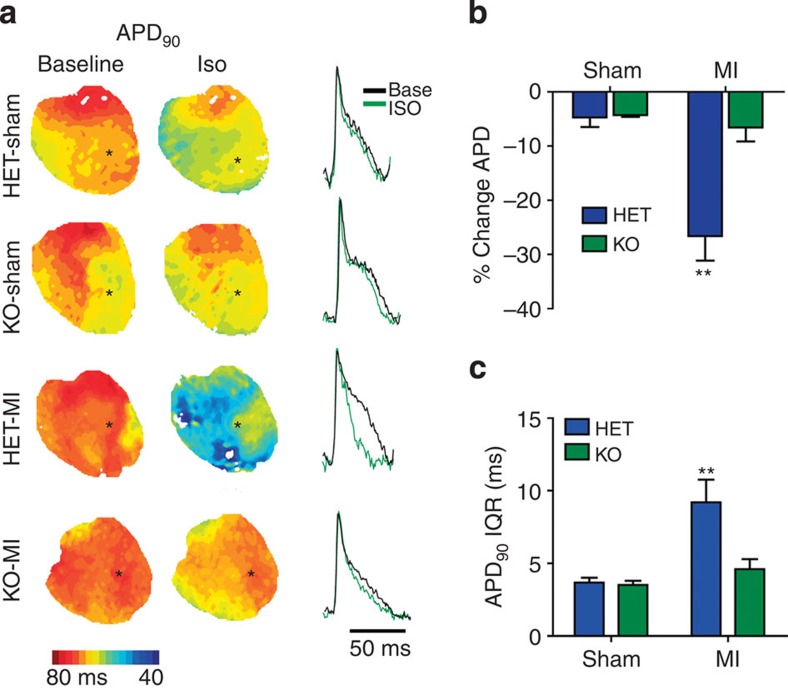
Sympathetic innervation of the infarct prevents β-AR supersensitivity. (**a**) APD_90_ maps before (Baseline) and after 1-μM ISO (100-ms pacing interval or 600 b.p.m. heart rate for each condition), and representative traces of optical action potentials from sites marked with *. Scale bar, 50 ms. (**b**) Percent change in APD_90_ following ISO treatment (mean±s.e.m.; *n*=4–5 hearts per group; **P<0.01 by two-way ANOVA with Bonferroni post test). (**c**) IQR of APD_90_ following ISO treatment (mean±s.e.m.; *n*=4–5 hearts; **P<0.01 by two-way ANOVA with Bonferroni post test).

Sudden cardiac death is most common in the morning^35^, when circulating catecholamines (NE and epinephrine) are rising rapidly^36^. Given the protective role of beta blockers in humans, and the observation that PVCs induced by the beta agonist ISO are a good marker of arrhythmia propensity in humans^25^, we examined the role of β-AR activation in arrhythmia generation in our infarcted mouse hearts. We treated hearts with tyramine to induce the release of endogenous NE from sympathetic axons within the heart, and with ISO to mimic circulating catecholamines. Release of endogenous NE with tyramine resulted in similar APD shortening across all groups (% change: HET Sham −0.5±0.6, HET MI −0.8±0.6, KO Sham −0.2±0.2, KO MI −2.1±0.6; *n*=3–5, mean±s.e.m.). In contrast, treatment with ISO, which mimics the effect of circulating catecholamines *in vivo*, stimulated a significantly greater shortening of APD_90_ in denervated HET MI hearts compared with all other groups (Fig. 4a,b). This denervation-induced β-AR supersensitivity is consistent with recent human data^21^. Consistent with the baseline data, APD_90_ dispersion (IQR) remained elevated in the HET MI hearts following ISO treatment (Fig. 4c).

### Sympathetic reinnervation prevents Ca^2+^ mishandling and PVCs

**Figure 5:**
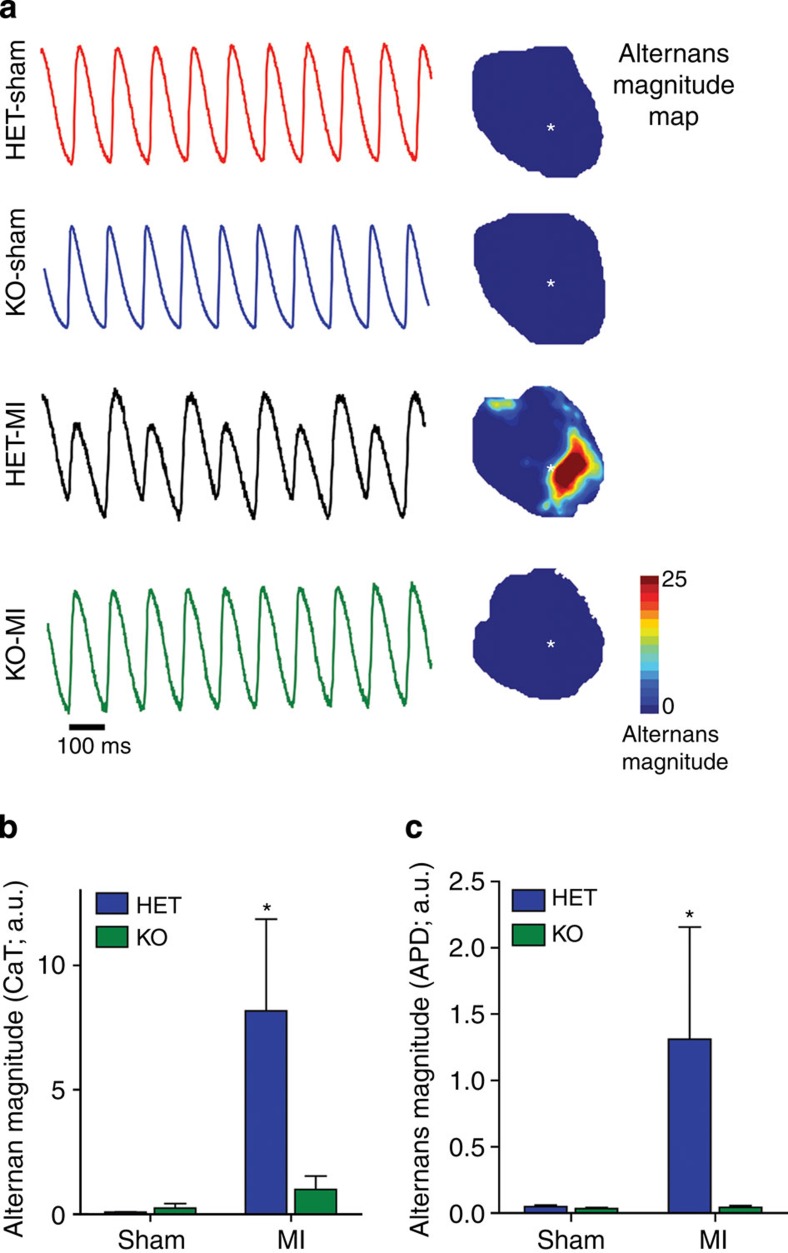
Sympathetic innervation of the infarct prevents post-MI Ca^2+^ mishandling. (**a**) Representative optical Ca^2+^ transients and alternans maps from the sites on sham and post-MI hearts marked (*) paced with a 100-ms pacing interval (600 b.p.m. heart rate). Scale bar, 100 ms. Denervated hearts (HET MI) exhibit Ca^2+^ alternans, while sham hearts and infarcted hearts with sympathetic innervation of the scar (KO MI) do not. (**b**) Intracellular Ca^2+^ alternans magnitude was quantified by spectral analysis (mean±s.e.m.; *n*=4–5 hearts; *P<0.05 versus other groups by two-way ANOVA with Bonferroni post test). (**c**) APD alternans magnitude was quantified by spectral analysis (mean±s.e.m.; *P<0.05 versus sham using Kruskal–Wallis test for non-Gaussian distribution).

In order to identify additional mechanisms underlying changes in cardiac electrophysiology, we examined intracellular Ca^2+^ handling at various pacing frequencies and after ISO treatment. Disrupted Ca^2+^ homeostasis can result in beat-to-beat fluctuations between large and small intracellular calcium transients (Ca_T_) called alternans, which is a sensitive noninvasive marker for the risk of sudden cardiac death in humans^37^. Intracellular Ca^2+^ handling appeared normal in sham hearts of both genotypes when paced with a 100-ms pulse interval (600 b.p.m. heart rate; Fig. 5). However, HET hearts with denervated infarcts (HET MI) exhibited significant intracellular Ca^2+^ alternans at the same pacing frequency (Fig. 5). Interestingly, KO hearts with innervated infarcts (KO MI) did not exhibit intracellular Ca^2+^ alternans (Fig. 5), suggesting that restoring sympathetic innervation to the infarct and surrounding tissue protects intracellular Ca^2+^ homeostasis. Denervated HET MI hearts also exhibited significant APD alternans (Fig. 5c), while innervated KO MI hearts did not, despite identical infarct size.

**Figure 6:**
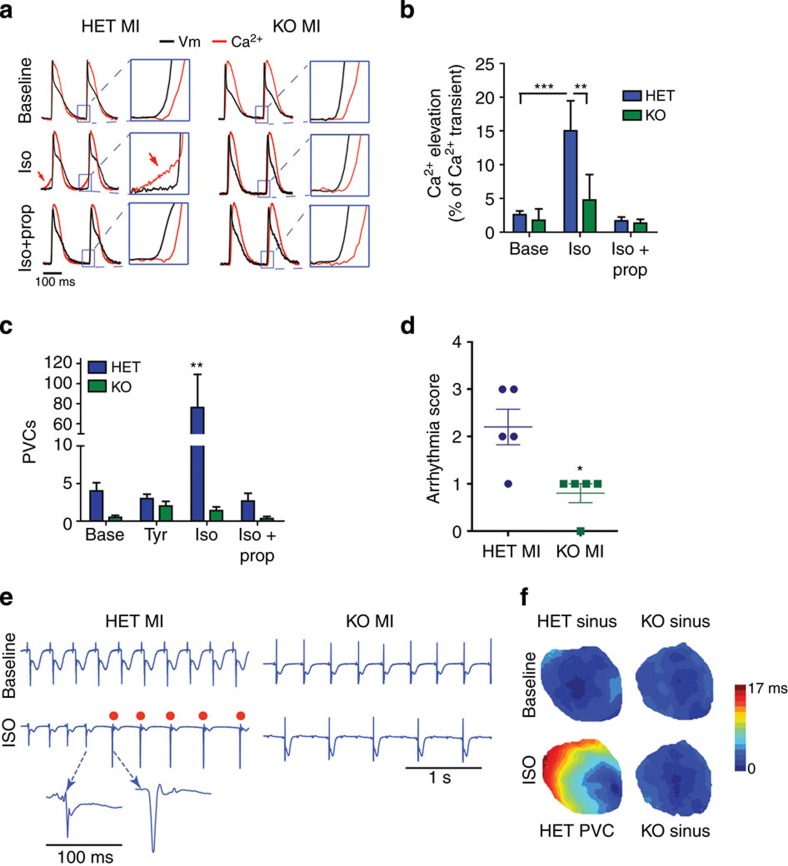
Reinnervation of the infarct prevents Ca^2+^ mishandling and ventricular arrhythmias. (**a**) Representative optical *V*_m_ and Ca^2+^ transients in HET and KO hearts at baseline (Base), with ISO, and with both ISO and propranolol (Iso+Prop). Red arrows indicate Ca^2+^ elevation before *V*_m_ depolarization. Scale bar, 100 ms. Insets show an expanded timescale of *V*_m_ and CaT upstrokes. (**b**) Quantification of CaT elevation that precedes depolarization averaged across the entire surface of the heart (mean±s.e.m.; *n*=4–5 hearts; **P<0.01, ***P<0.001 by two-way ANOVA with Bonferroni post test). (**c**) Incidence of PVCs during baseline stimulation and treatment with tyramine (Tyr), ISO (Iso) and ISO plus propranolol (Iso+Prop; mean±s.e.m.; *n*=4–5 hearts; **P<0.01 versus all other groups by two-way ANOVA with Bonferroni post test). (**d**) Arrhythmia severity of HET MI and KO MI hearts during ISO treatment. Individual hearts are scored on the basis of the most severe arrhythmia observed (mean±s.e.m.; *n*=4–5 hearts; *P<0.05 by *t*-test). (**e**) Example Lead I ECGs from *in vitro* Langendorff-perfused hearts during baseline and with ISO. Owing to ISO-induced acceleration of the sinus rate, hearts in both groups exhibited atrioventricular (A–V) block (see P-wave dissociation and 3:1 A-V block in ISO traces); however, PVCs were significantly more frequent in HET MI hearts (red dots). PVCs were readily identified by a large and wide QRS complex compared with the normal (sinus or nodal) QRS complex (inset). (**f**) Representative activation maps in HET MI and KO MI hearts depicting rapid activation during sinus rhythm (Base line) in both hearts and with ISO treatment (Iso) in the KO MI heart. Iso treatment in the denervated (HET MI) heart produced PVCs with slowly propagating activation arising from the infarct region.

The notion that sympathetic reinnervation of the peri-infarct border and scar protects intracellular Ca^2+^ handling was confirmed by analysing intracellular Ca^2+^ dynamics after ISO administration. Under baseline conditions, normal excitation–contraction coupling occurred, in which membrane depolarization (*V*_m_; black) preceded the Ca_T_ upstroke (red) in hearts from both genotypes and surgical groups (Fig. 6a, inset). In sham hearts treated with ISO, depolarization continues to precede the Ca_T_ (Supplementary Fig. 3). However, in the infarcted hearts, the response to ISO differed depending on the innervation status. Infarcted hearts with sympathetic innervation restored throughout the entire left ventricle (KO MI) continued to exhibit normal Ca^2+^ handling after ISO treatment (Fig. 6a,b), while hearts with sympathetic denervation (HET MI) exhibited pathological increases in diastolic intracellular [Ca^2+^] that preceded membrane depolarization (Fig. 6a,b). This Ca^2+^ elevation was prevented by addition of the β-AR blocker propranolol (Fig. 6a,b).

We previously showed that strong localized β-AR stimulation can trigger Ca^2+^ release independent of membrane depolarization that is sufficient to cause PVCs^38^. Therefore, we asked whether ISO-stimulated Ca^2+^ mishandling led to the production of PVCs after MI. Quantification of *in vitro* arrhythmia severity in HET and KO MI hearts confirmed the striking difference in arrhythmia susceptibility observed during the *in vivo* ECG telemetry studies. Arrhythmia severity was scored on the basis of the most severe arrhythmia observed in each heart over a 20-min continuous ECG recording (0=no PVCs, 1=single PVCs, 2=bigeminy or salvos and 3=ventricular tachycardia)^39^. Sham animals of both genotypes exhibited only single PVCs, and ISO stimulated a similar number in both genotypes (HET versus KO; Base: 1.00±0.71 versus 1.33±0.88 and Iso: 1.75±0.48 versus 1.33±0.33). ISO triggered significantly more arrhythmias in HET MI hearts with denervated infarct/peri-infarct tissue compared with all other groups (Fig. 6c,e), and it stimulated arrhythmias of greater severity in those hearts, including ventricular tachycardia (Fig. 6d). In contrast, KO MI hearts with innervated infarct/peri-infarct tissue exhibited only individual PVCs and the number of PVCs was similar to that seen in the sham groups (Fig. 6c), even though infarct size was comparable to HET MI hearts. As expected, administration of propranolol prevented ISO-induced arrhythmias in HET MI hearts, confirming the role of β-AR (Fig. 6c). Importantly, Ca^2+^ mapping revealed that the Ca^2+^ elevation induced by ISO was associated with PVCs arising from the infarct region (Fig. 6f). All PVCs that were captured during a mapping procedure arose from the region of the infarct, suggesting that denervation-induced β-AR supersensitivity leads to diastolic Ca^2+^ elevation near the infarct that is sufficient to trigger PVCs. Restoration of sympathetic innervation to the infarct and surrounding tissue normalizes Ca^2+^ handling and makes hearts resistant to ISO-induced arrhythmias.

## Discussion

The observation that local sympathetic denervation in the post-MI heart leads to high arrhythmia risk seems to contradict the many clinical studies showing that blocking sympathetic transmission in the heart, either with beta blockers or with surgical ganglionectomy, prolongs life after MI^3^,^6^,^7^,^40^,^41^. However, a consensus has developed that inappropriate heterogeneity of sympathetic transmission in the heart, and subsequent electrical remodelling of cardiac myocytes, is arrhythmogenic^22^. This heterogeneity can be caused by the loss of sympathetic transmission in a portion of the heart^18^,^19^,^20^,^21^, or it can be caused by regional nerve sprouting and hyperinnervation^42^,^43^,^44^,^45^,^46^. In this context, surgical denervation and administration of beta blockers can be seen as methods for ‘evening out’ sympathetic transmission across the heart by decreasing it everywhere. Our data fully support the model that heterogeneity of sympathetic transmission after MI is pathological; however, our approach to correct that problem was to restore sympathetic transmission throughout the left ventricle by targeting PTPσ. It is especially notable that we were able to restore innervation using a pharmacologic intervention that was initiated several days after injury when the infarct and peri-infarct myocardium was already denervated. Daily injections with ISP were sufficient to restore functional sympathetic innervation, and reinnervation rendered hearts surprisingly resistant to arrhythmias. Indeed, infarcted hearts with restored sympathetic innervation were electrically indistinguishable from sham hearts, despite the presence of a scar.

This work has implications for patients who survive a MI, and remain at risk for severe cardiac arrhythmias and sudden cardiac death^1^. Those patients who have a left ventricular (LV) ejection fraction ≤35% often receive prophylactic implantation of an ICD^47^; however, low ejection fraction has not proven to be a good predictor of those truly in need of ICDs^48^. Substantial effort has gone into identifying parameters that can predict those patients most likely to benefit from ICDs, and three recent trials found that sympathetic denervation within the heart after MI predicted the incidence of sudden cardiac arrest and arrhythmia independent of infarct size and LV ejection fraction^18^,^19^,^20^. Our study provides a mechanistic link between post-infarct denervation and altered beta receptor responsiveness, calcium handling and arrhythmia susceptibility.

There are limitations, however, to the mouse model for studies of cardiac rhythm disturbances, as the repolarizing currents in the rodent heart are different from those in larger species^32^,^49^. In addition, the scar formed in the mouse heart contains less collagen than the scar in larger animals^30^, and that might have an impact on arrhythmia susceptibility. Other limitations of our experimental model are not unique to the mouse. For example, we used ISO or electrical stimulation protocols to trigger arrhythmias because spontaneously occurring arrhythmias were relatively rare events in our mice. However, arrhythmias are rare even in patients with significant LV systolic dysfunction following MI, exemplified by the fact that it takes many years to demonstrate benefit from an ICD in such populations^48^. Thus, interventions including exercise stress testing^50^ or ISO^25^ are used by clinicians to evoke arrhythmias in the clinic, and PVCs triggered by these methods are indicative of arrhythmia propensity in humans. Therefore, our data suggest a potential site for therapeutic intervention, but future studies are necessary to test the anti-arrhythmic efficacy of ISP in larger animal models.

These data provide an important validation of the hypothesis that targeting PTPσ can promote nerve regeneration through CSPG-containing scars *in vivo*, and suggest that reversing infarct-induced sympathetic denervation is protective for the heart. MI is an ideal target for PTPσ-based therapeutics because the cardiac scar contains high levels of nerve growth factor and simply removing PTPσ is sufficient to promote reinnervation of the infarct^23^. However, these data also have important implications for the treatment of spinal cord injury and other peripheral nerve injuries where CSPG-PTPσ interactions prevent nerve regeneration. Systemic injection of the peptide ISP was effective in restoring axon regeneration through CSPGs in the cardiac scar and decreasing arrhythmia risk. This raises the possibility that targeting PTPσ using a simple systemically deliverable peptide in patients who have survived a MI might promote reinnervation of otherwise denervated cardiac tissue, and that normalizing the innervation might decrease arrhythmia risk.

## Methods

### Animals

PTPσ (ptprs) transgenic mice (BalbC) were supplied by Michel Tremblay (McGill University)^51^ and were bred as heterozygotes^23^. Age- and gender-matched mice that were 12–18 weeks old were used for all experiments. ptprs+/− (HET) littermates were used as controls for ptprs−/− (KO) studies. Some ptprs+/+ mice were given daily IP injections of vehicle (5% DMSO/Saline), IMP (Intracellular Mu Peptide; 10 μmol) or ISP (10 μmol) beginning 3 days after I–R. All mice were kept on a 12:12 h light–dark cycle with ad libitum access to food and water. All procedures were approved by the OHSU and the University of California-Davis Institutional Animal Care and Use Committee and comply with the Guide for the Care and Use of Laboratory Animals published by the National Academies Press (8th edition).

### Surgery

(1) *Myocardial I–R*: Anaesthesia was induced with 4% isoflurane and maintained with 2% isoflurane. The left anterior descending coronary artery was reversibly ligated for 30 min (telemetry studies) or 45 min (*ex vivo* mapping) and then reperfused by release of the ligature^23^,^24^. Occlusion was confirmed by sustained S–T wave elevation and regional cyanosis. Reperfusion was confirmed by the return of colour to the ventricle distal to the ligation and reperfusion arrhythmia. Core body temperature was monitored by a rectal probe and maintained at 37 °C, and a two-lead ECG was monitored. (2) *Sham surgery*: Sham animals underwent the procedure described above, except for the left anterior descending coronary artery ligature.

### *In vivo* telemetry

ECGs were obtained from conscious adult mice using ETA-F10 (Data Sciences International) telemetry implants, and were analysed with the Dataquest ART software (Data Sciences International). Mice were anaesthetized with 4% and maintained on 2% inhaled isoflurane. A transmitter was implanted subcutaneously in a lead II configuration, with the negative lead placed in the right pectoral muscle and the positive lead to the left of the xyphoid process. Devices were implanted at least 5 days before I–R or sham surgery^12^.

ECG recordings were obtained 10 (PTPσ transgenic studies) or 14 (ISP studies) days after sham or MI surgery. ECGs were analysed beginning 60 min before IP injection of a relatively low dose of the β-AR agonist ISO (10 μg or ~0.5 mg kg^−1^)^52^,^53^ as a baseline, and then for 60 min after injection to identify ISO-induced arrhythmias. PVCs were defined as a single premature QRS complex in the absence of a P-wave. Heart rate was analysed to confirm that the SA node response to ISO was similar between groups.

### Immunohistochemistry

Hearts were fixed for 1 h in 4% paraformaldehyde, rinsed in PBS, cryoprotected in 30% sucrose overnight and frozen in mounting medium for sectioning. Transverse 10-μm sections were cut on a cryostat and thaw-mounted on charged slides. To reduce fixative-induced autofluorescence, sections were rinsed in 10 mg ml^−1^ sodium borohydride for 3 × 10 min and then rinsed in PBS for 3 × 10 min. The sections were then blocked in 3% BSA/0.3% Triton X-100 in PBS for 1 h. The slides were then incubated with rabbit anti-TH (1:1,000, Millipore/Chemicon) and sheep anti-fibrinogen (1:500, AbD Serotec) antibodies overnight at 4 °C, rinsed for 3 × 10 min in PBS and incubated for 1.5 h with the AlexaFluor 488-conjugated rabbit IgG-specific antibody (1:1,000; Invitrogen) and AlexaFluor 568-conjugated sheep IgG-specific antibody (1:1,000). Sections were rinsed for 3 × 10 min in PBS, incubated for 30 min in 10 mM CuSO_4_ in 50 mM ammonium acetate to reduce background signal further, rinsed for 3 × 10 min in PBS, coverslipped in a 1:1 PBS:glycerol solution and visualized using fluorescence microscopy.

### Imaging and threshold analysis

TH staining was quantified to assess sympathetic innervation density. Images were taken of the infarct, proximal and distal peri-infarct zone of each section, and three sections obtained from a similar level of the base-to-apex axis were quantified in each heart using threshold discrimination analysis. Black and white photos were opened in ImageJ and the brightness/contrast tool was used to adjust each image so that the minimum was set at the left side of the histogram. The threshold tool was then used to identify nerve fibres. The automated function set a beginning threshold level; however, this did not reliably include all sympathetic fibres while excluding all non-neuronal tissues. The threshold was then manually adjusted to ensure that only specific TH staining was identified. The ventricle has two layers of muscle set at different angles; therefore, each section included some nerve fibres cut longitudinally along the plane of the section and other fibres cut in cross-section and appearing as small round dots. Thus, specific criteria for size and shape were not used in our analysis. Innervation density was expressed as the percent of area that was above the threshold (TH+ fibres). Each image was quantified by two independent observers.

### Infarct size

Infarcts were identified with fibrinogen staining and infarct size was quantified using eight 10-μm sections, ~200 μm apart obtained from matched levels from each heart. Images were taken of the entire section, and the LV and infarct areas were measured by outlining the respective regions using the freehand selection tool in ImageJ. Infarct size was then calculated as a percentage of the LV area [(infarct area/LV area) × 100]. Sections were obtained from the upper, middle and lower regions of the infarct in each heart.

### NE content

NE levels in heart tissue were measured using HPLC with electrochemical detection. Frozen, pulverized tissue was weighed, and then homogenized in 0.2 M perchloric acid and 0.5 μM dihydroxybenzylamine (DHBA). The tissue was refrigerated for 1 h and centrifuged at 14,000 r.p.m. for 4 min. Supernatant was adsorbed on alumina, followed by 15 min of tumbling. The alumina was washed twice with 1.0 ml of H_2_O with centrifugations in between. The NE was desorbed from the alumina with 150 μl of 0.1 M perchloric acid. Fifty-microlitre aliquots were fractionated by reversed-phase HPLC (C18; 5 μm particle size, Rainin) using a mobile phase containing 75 mM sodium phosphate (pH 3.0), 360 mg l^−1^ of sodium octane sulfonate, 100 μl l^−1^ triethylamine and 3.0% acetonitrile. A coulometric detector (Coulchem, ESA) was used to detect and quantify NE and DHBA. NE standards (0.5 μM) were processed in parallel with tissue samples and interspersed throughout the HPLC run. Retention time for NE was 5.0 min and for DHBA was 8.5 min.

### Langendorff perfusion and dual optical mapping of *V*_m_ and Ca^2+^

Mice were anaesthetized with pentobarbital sodium (150 mg kg^−1^, IP) containing 120 IU of heparin. Following a midsternal incision, hearts were rapidly excised and Langendorff-perfused at 37 °C with oxygenated (95% O_2_ and 5% CO_2_) modified Tyrode’s solution of the following composition (in mmol l^−1^): NaCl 128.2, CaCl_2_ 1.3, KCl 4.7, MgCl_2_ 1.05, NaH_2_PO_4_ 1.19, NaHCO_3_ 20 and glucose 11.1 (pH 7.4). Flow rate (1.5–3.5 ml min^−1^) was adjusted to maintain a perfusion pressure of 60–80 mm Hg. One leaflet of the mitral valve was carefully damaged with sharp forceps inserted through the pulmonary vein to prevent solution congestion in the LV cavity after suppression of ventricular contraction. Two Ag/AgCl disc electrodes were positioned in the bath to record an ECG analogous to a lead I configuration. ECG was continuously recorded throughout the duration of the experiment. A bipolar electrode was positioned on the base of the LV epicardium for pacing, which was performed at a basic cycle length of 150 ms using a 2-ms pulse width at twice the diastolic threshold.

Hearts were loaded with the fluorescent intracellular Ca^2+^ indicator Rhod-2 AM (Molecular Probes, Eugene, OR; 50 μl of 1 mg ml^−1^ in DMSO containing 10% pluronic acid) and were subsequently stained with the voltage-sensitive dye RH237 (Molecular Probes; 10 μl of 1 mg ml^−1^ in DMSO). Blebbistatin (Tocris Bioscience, Ellisville, MO; 10–20 μM) was added to the perfusate to eliminate motion artifact during optical recordings. The anterior epicardial surface was excited using LED light sources centred at 530 nm and bandpass-filtered from 511 to 551 nm (LEX-2, SciMedia, Costa Mesa, CA) and focused directly on the surface of the preparation. The emitted fluorescence was collected through a 50-mm objective (Nikon, Japan) and split with a dichroic mirror at 630 nm (Omega, Brattleboro, VT). The longer wavelength moiety, containing the *V*_m_ signal, was longpass-filtered at 700 nm and the shorter wavelength moiety, containing the Ca^2+^ signal, was bandpass-filtered between 574 and 606 nm. The emitted fluorescence signals were recorded using two CMOS cameras (MiCam Ultima-L, SciMedia) with a sampling rate of 1 kHz and 100 × 100 pixels with a 10 × 10 mm field of view^38^.

### Mapping experimental protocol

Following dye loading, baseline electrophysiological parameters were recorded during normal sinus rhythm as well as LV epicardial pacing at intervals of 150 and 100 ms. Hearts were subjected to tyramine infusion (20 μM) followed by a washout period. Hearts were then subjected to acute β-AR stimulation with ISO (1 μM). Optical recordings were taken every 5 min following treatment, and ECG was continuously recorded. Following 15 min of ISO treatment, the β-AR blocker propranolol (10 μM) was added to the perfusate. Optical recordings were repeated every 5 min for 15 min.

### Optical mapping data analysis

Optical mapping data analysis was performed using two commercially available analysis programmes (BV_Analyze, Brainvision, Tokyo, Japan and Optiq, Cairn, UK). *V*_m_ and Ca^2+^ data sets were spatially aligned and processed with a Gaussian spatial filter (radius 3 pixels). For both action potentials and Ca_T_, activation time was determined as the time at 50% of the maximal amplitude. For action potentials, repolarization time at 30 and 90% return to baseline were used to calculate APD (APD_30_ and APD_90_). Relative APD_30_ and APD_90_ used in restitution curves were calculated as a ratio of S2 APD/S1 APD. Conduction velocity was calculated along a vector from the base to apex using a polynomial fitting algorithm previously described in ref. 54. Separate measurements of longitudinal and transverse conduction velocity were not determined because of the basal pacing location and disruption of fibre orientation in the infarct region. Diastolic Ca^2+^ elevation was measured as the percentage of diastolic Ca^2+^ increase relative to the following Ca_T_ amplitude at baseline and 15 min post treatment. The average diastolic Ca^2+^ elevation was calculated for each heart by averaging all Ca^2+^ signals from the entire anterior surface of the heart within the optical mapping field of view. PVC incidence was determined from the continuous ECG recording as the number of PVCs that occurred during a 20-min period of baseline activity (before initiation of treatment) and during the 20 min of treatment. Arrhythmia severity was scored on the basis of the most severe arrhythmia observed in each heart over a 20-min continuous ECG recording (0=no PVCs, 1=single PVCs, 2=bigeminy or salvos and 3=ventricular tachycardia)^39^.

The spectral method, which has been used clinically for detecting micro-volt T-wave alternans^55^, was used to detect the presence of Ca_T_ alternans and APD alternans^56^. The spectral method was chosen because of its high sensitivity and relative immunity to noise. This approach allowed us to determine whether an area within the mapping field of view was experiencing significant APD or Ca_T_ alternans (greater than the background noise levels) as well as the spatial extent of significant alternans. A spectral magnitude of ≥2 was used as the minimum threshold for significant APD or Ca_T_ alternans, corresponding to a beat-to-beat change in APD_90_ ≥5 ms or beat-to-beat change in Ca_T_ amplitude ≥5%, respectively^57^.

### Statistics

Student’s *t*-tests were used to compare two groups. Data sets that included more than two groups were analysed by one-way analysis of variance (ANOVA) using the Tukey *post hoc* test to compare all conditions. For experiments comparing different surgical groups and genotypes a two-way ANOVA was carried out using the Bonferroni *post hoc* test. All statistical analyses were carried out using Prism 5.0.

## Additional information

**How to cite this article:** Gardner, R. T. *et al.* Targeting protein tyrosine phosphatase σ after myocardial infarction restores cardiac sympathetic innervation and prevents arrhythmias. *Nat. Commun.* 6:6235 doi: 10.1038/ncomms7235 (2015).

## Supplementary Material

Supplementary Information Supplementary Figures 1-3.
