# Supplementary material for: Targeting protein tyrosine phosphatase σ after myocardial infarction restores cardiac sympathetic innervation and prevents arrhythmias
Source: Nat Commun. 2015 Feb 2;6:6235. doi: 10.1038/ncomms7235 (PMC4315356; doi:10.1038/ncomms7235)
Supplement: Supplementary Information — Supplementary Figures 1-3. [file ncomms7235-s1.pdf]

Supplementary Figures

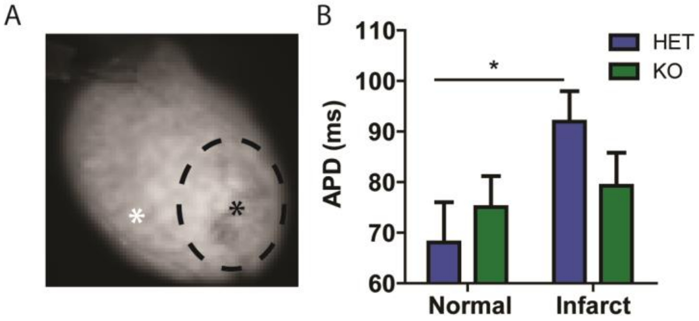

Supplementary Figure 1. Regional variation in APD following MI. (A) An image of a heart prepared for mapping. The dotted area identifies the approximate location of infarcted myocardium. The black asterisk represents the location where APD was measured for the infarct, while the white asterisk identifies where APD was measured for normal, un-injured myocardium. **(B)** Quantification of regional APD. HET hearts have a significantly longer APD within the infarct compared to normal tissue. The reinnervated KO hearts, however, have similar APD in both regions. The regional differences observed in HET hearts explain the greater dispersion of APD (as measured by IQR). Data are mean ± SEM, n=5 hearts/genotype. *p<.05 using a two-way ANOVA and Bonferroni post-test.

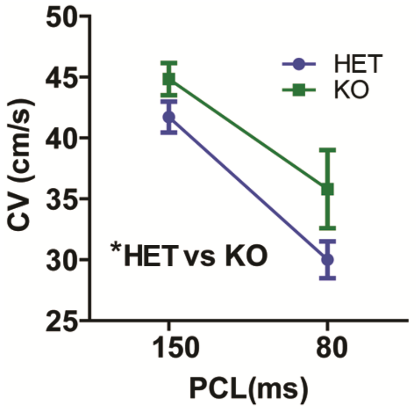

Supplementary Figure 2. Conduction velocity (CV) following MI. As expected, changing the pacing interval from 150 ms (400 bpm) to 80 ms (750 bpm) results in a rate-dependent slowing of CV in both HET and KO hearts. KO hearts, however, have significantly faster CV overall. Data are mean ± SEM, n=5 hearts/genotype. * $p<.05$ using a two-way ANOVA and Bonferroni post-test.

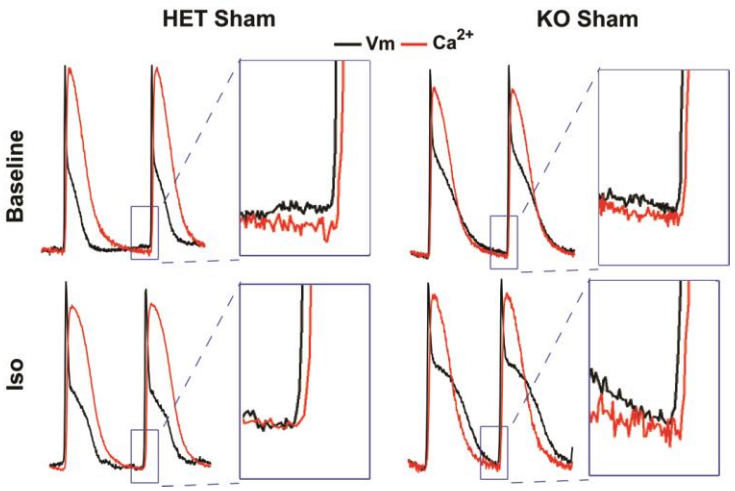

Supplementary Figure 3. Ca^{2+} handling is normal in sham hearts. Representative optical Ca^{2+} transients (Ca_T) in HET and KO sham hearts at baseline, and with isoproterenol (Iso). Note that the Ca_T upstroke follows V_m depolarization even in the presence of Iso. Insets show an expanded time scale of V_m and Ca_T upstrokes.
